# Short Telomere Lesions with Dysplastic Metaplasia Histology May Represent Precancerous Lesions of *Helicobacter pylori*-Positive Gastric Mucosa

**DOI:** 10.3390/ijms24043182

**Published:** 2023-02-06

**Authors:** Rina Fujiwara-Tani, Tadataka Takagi, Shiori Mori, Shingo Kishi, Yukiko Nishiguchi, Takamitsu Sasaki, Masayuki Ikeda, Kenta Nagai, Ujjal Kumar Bhawal, Hitoshi Ohmori, Kiyomu Fujii, Hiroki Kuniyasu

**Affiliations:** 1 Department of Molecular Pathology, Nara Medical University, 840 Shijo-cho, Kashihara 634-8521, Japan; 2 Miyoshi Central Hospital, 10531 Higashi-Sakaya-cho, Miyoshi 728-8502, Japan; 3 Department of Biochemistry and Molecular Biology, Nihon University School of Dentistry at Matsudo, Matsudo 271-8587, Japan; 4 Department of Pharmacology, Saveetha Institute of Medical and Technical Sciences, Saveetha Dental College, Chennai 600077, India

**Keywords:** gastric cancer, precancerous lesion, *H. pylori*, stemness, telomere

## Abstract

Gastric cancers are strongly associated with *Helicobacter pylori* infection, with intestinal metaplasia characterizing the background mucosa in most cases. However, only a subset of intestinal metaplasia cases proceed to carcinogenesis, and the characteristics of high-risk intestinal metaplasia that link it with gastric cancer are still unclear. We examined telomere reduction in five gastrectomy specimens using fluorescence in situ hybridization, and identified areas with localized telomere loss (outside of cancerous lesions), which were designated as short telomere lesions (STLs). Histological analyses indicated that STLs were characteristic of intestinal metaplasia accompanied by nuclear enlargement but lacking structural atypia, which we termed dysplastic metaplasia (DM). A review of gastric biopsy specimens from 587 *H. pylori*-positive patients revealed 32 cases of DM, 13 of which were classified as high-grade based on the degree of nuclear enlargement. All high-grade DM cases exhibited a telomere volume reduced to less than 60% of that of lymphocytes, increased stemness, and telomerase reverse transcriptase (TERT) expression. Two patients (15%) exhibited low levels of p53 nuclear retention. After a 10-year follow-up, 7 (54%) of the high-grade DM cases had progressed to gastric cancer. These results suggest that DM is characterized by telomere shortening, TERT expression, and stem cell proliferation, and high-grade DM is a high-grade intestinal metaplasia that likely represents a precancerous lesion of gastric cancer. High-grade DM is expected to effectively prevent progression to gastric cancer in *H. pylori*-positive patients.

## 1. Introduction

Gastric cancer accounts for approximately 6% of all cancers worldwide and is the fifth most commonly diagnosed malignancy, as well as being the third leading cause of cancer-related death [1]. Ninety percent of noncardia gastric cancers are associated with *Helicobacter pylori* infection, and its eradication suppresses malignant transformation [2]. Chronic active inflammation due to *H. pylori* infection causes histologically atrophic gastritis and intestinal metaplasia [3,4]. Intestinal metaplasia has been considered to be a precancerous lesion of gastric cancer [5], and patients with this phenotype have more than 10-fold increased risk of developing gastric cancer than those with non-metaplastic mucosa [6]. However, the carcinogenic rate of intestinal metaplasia is only 0.09–1.7% per year and it appears to represent a precancerous state rather than a precancerous lesion [7,8]. The characterization of high-risk intestinal metaplasia, which is a true precancerous lesion that progresses from intestinal metaplasia to cancer, may fill a missing piece in our understanding of the development of *H. pylori*-associated gastric cancer. Identifying the characteristics of precancerous lesions that lead to carcinogenesis may also reveal avenues for preventing gastric cancer.

Using fluorescence in situ hybridization (FISH), we have previously demonstrated that *H. pylori* infection leads to telomere shortening in the gastric epithelium [9]. Telomere shortening and telomerase reverse transcriptase (TERT) activation are frequently observed in gastric cancer and are considered to be early events in carcinogenesis of the gastric mucosa [10,11]. TERT activity is a phenotype observed in stem cells in normal tissues [12], and telomere shortening triggers aberrant activation of telomerase [13]. The mechanisms underpinning the loss of TERT gene repression involve oncogenic factors, signal transduction dysregulation, post-transcriptional/post-translational regulation, genomic amplification, and promoter mutation [14]. In gastric cancer, TERT promoter mutation is a rare event [14], and TERT expression is observed in gastric epithelium with markedly decreased telomeres [9]. This suggests that the repeated regeneration and proliferation associated with *H. pylori* infection in gastric cancer may cause telomere shortening, resulting in induction of TERT expression, telomerase activation, and an increase in stem cells. Thus, stem cell hyperplasia has been suggested as an early event in *H. pylori*-associated carcinogenesis [15,16]. In addition, mTOR/AKT activation has been observed in high-risk gastric mucosa and correlates with TERT expression and stem cell proliferation [17,18,19]. As telomere reduction is considered to be an early event of gastric carcinogenesis, we hypothesized that it may be a feature of high-risk lesions. The aim of this study was therefore to characterize high-risk lesions in intestinal metaplasia using telomere reduction as a marker, with a view to identifying the molecular state of metaplasias that proceed to gastric cancer.

## 2. Results

### 2.1. Short Telomere Lesions in Gastric Cancer Cases

**Figure 1:**
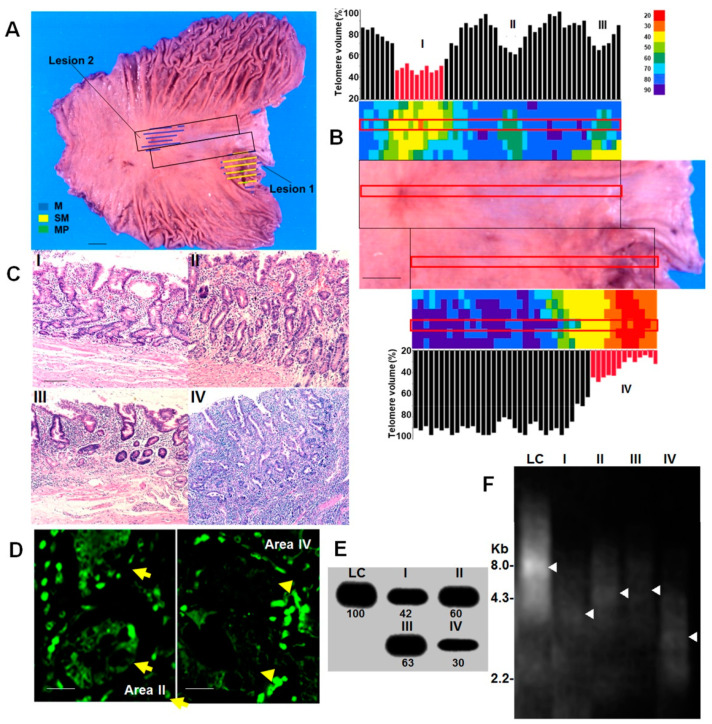
Short telomere lesion in gastric mucosa—Case 1. (**A**) Macroscopic image of mucosal aspect of the stomach with two gastric cancer lesions (Case 1 in Table 1). Scale bar, 1 cm. Boxes, TV examined areas showed in panel B, Color indicator, invasive depth and area. (**B**) TV of the gastric mucosa containing both gastric lesions. The heat map shows a geographical image of TV, and the bar graphs display the TV at the respective points in the area within the red box. Scale bar, 1 cm. (**C**) HE-stained tissue from areas I–IV indicated in panel B. Scale bar, 100 μm. (**D**) Telomere FISH in area II and IV. Arrow, metaplastic glands. Arrow head, cancer galnds. Scale bar, 50 μm. (**E**) TV by slot blot hybridization using (TTAGGG)_4_ probe. LC, lymphocytes. Number, semi-quantified density. (**F**) Telomere length via Southern blot hybridization using (TTAGGG)_4_ probe. LC, lymphocytes. Arrow head, peak density. M, mucosal layer; SM, submucosal layer; MP, muscularis propria layer; TV, telomere volume; HE, hematoxylin & eosin staining; FISH, fluorescent in situ hybridization.

**Figure 2:**
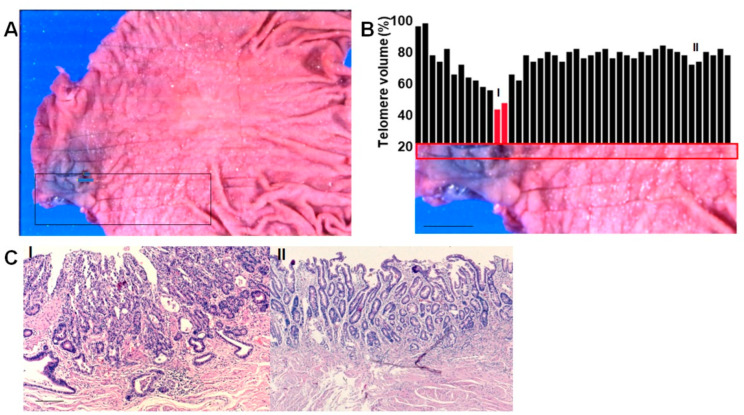
Short telomere lesion in gastric mucosa—Case 3. (**A**) Macroscopic image of mucosal aspect of the stomach with two gastric cancer lesions (Case 3 in Table 1). Scale bar, 1 cm. Boxes, TV examined areas showed in panel B, Color indicator, invasive depth and area. (**B**) TV of the gastric mucosa. Bar graphs show TV at the respective points in the area within the red box. Scale bar, 1 cm. (**C**) HE-stained tissue from areas I–II indicated in panel (**B**). Scale bar, 100 μm. M, mucosal layer; TV, telomere volume; HE, hematoxylin & eosin staining.

Using FISH, we first examined telomere volume (TV) in five cases of gastrectomy due to gastric cancer (Table 1, Figure 1 and Figure 2). In case 1, advanced cancer was found in the cardia and early cancer in the antrum (Figure 1A). TV was significantly decreased in the cancer cells, and in the peritumoral mucosa, epithelial TV decreased closer to the cancer site (Figure 1B). TV also decreased to 60–66% in non-cancerous mucosa that was not continuous with the cancer lesion, and the heat map showed a localized distribution of low-TV epithelium (areas II and III). 

When we examined histological images of the cancer and the non-cancerous mucosa with decreased TV (Figure 1C), we observed that cancer lesion 1 (area IV) was more atypical than cancer lesion 2 (area I), with a decreased TV. Area II exhibited intestinal metaplasia, and nuclear enlargement that was more than twice the size of the lymphocyte nuclei was observed in more than 1/3 of the lower portion of the gland, with no structural atypia. In contrast, in area III, nuclear enlargement less than twice that of the lymphocyte nuclei was observed within the lower 1/3 of the glands, and no structural atypia was observed.

When TV was evaluated by slot blot for areas I–IV, all regions showed a decrease in TV equivalent to that detected by FISH (Figure 1D). Furthermore, Southern blot analysis showed that telomeres were 8 kb in lymphocytes, but 4 and 3 kb in areas I and IV (the cancer lesions), respectively. In focal telomere-depleted areas II and III, telomeres were 4.3 kb and 4.5 kb, respectively. Thus, TVs assessed by FISH and slot blotting correlated well with telomere lengths ascertained by Southern blot analysis.

In case 3, a cancer lesion was observed in the antrum prepylorus, as shown in Figure 2A. A decrease in TV (47%) was observed in the cancer lesion (Figure 2B, area I). In addition, a focal reduction in TV was also seen in the non-cancerous mucosa, with a TV of 72% (area II). Examination of the tissue in each area revealed that the cancer could be classified as tub1 (well-differentiated tubular adenocarcinoma), remaining within the mucosa (Figure 2C). Area II showed intestinal metaplasia, and nuclear enlargement less than twice that of lymphocyte nuclei was observed within the lower 1/3 of the glands, with no structural atypia.

All five gastric cancer cases were positive for *H. pylori* and a marked decrease in TV was observed in the cancer lesions (Table 1). A decrease in TV was also observed in the peritumoral mucosa. In addition, focal TV reduction was observed in all cases, and two of these lesions had nuclear enlargement greater than twice the size of the lymphocyte nuclei in more than the lower 1/3 of the gland.

### 2.2. Morphological Features of Short Telomere Lesions 

As mentioned above, focal TV reduction was observed around cancer lesions and in non-cancerous areas, accompanied by nuclear enlargement. Based on these results, we named lesions with localized TV reduction “short telomere lesions” (STLs). STLs are characterized by the histological features of nuclear enlargement, decreased mucus production, and goblet cell depletion, and were designated as dysplastic metaplasia (DM). Based on these histological characteristics, STLs were classified into high-grade and low-grade according to the criteria shown in Table 2. In addition, STLs with nuclear enlargement more than twice the size of the lymphocyte nuclei in more than 1/3 of the basal side of the glands were classified as high-grade DM.

### 2.3. Extraction of DM Cases

**Figure 3:**
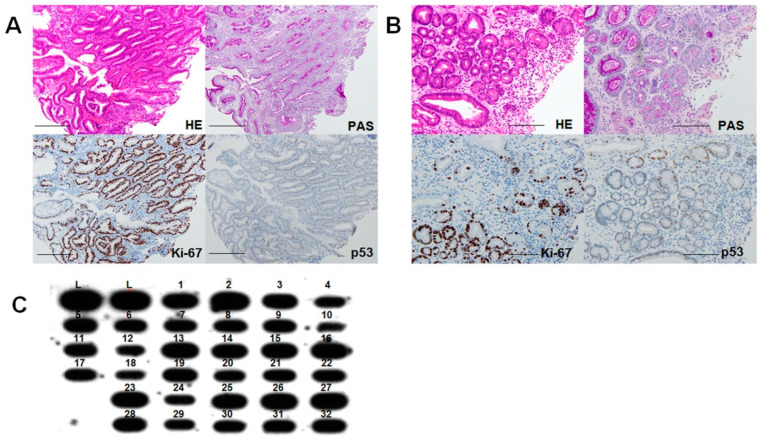
Dysplastic metaplasia in gastric mucosa. (**A**) Histological appearance of low-grade DM (Case 1 in Table 3). (**B**) Histological appearance of high-grade DM (Case 12 in Table 3). (**C**) TV by slot blot hybridization using (TTAGGG)_4_ probe. HE, hematoxylin & eosin staining; PAS, periodic acid-Schiff staining; DM, dysplastic metaplasia; TV, telomere volume; L, lymphocytes.

Next, we histologically reviewed 587 *H. pylori*-positive gastric mucosal biopsies diagnosed between 2005 and 2015. Our analyses revealed 32 cases of DM consistent with the morphological characteristics outlined in Table 2 (Table 3). In the DM tissues, mucus secretion was reduced compared with the surrounding normal intestinal metaplasia, and there was an increase in the number of cells positive for TERT and nucleostemin (NS) (a stem cell marker) (Figure 3A,B). Low levels of p53 expression were also observed in two cases of high-grade DM (Figure 3B). When we measured TV by slot blotting in the DM tissues, the results showed that TV was decreased in all cases compared to normal lymphocytes (Figure 3C).

### 2.4. Phenotypes of High-Grade DM

**Figure 4:**
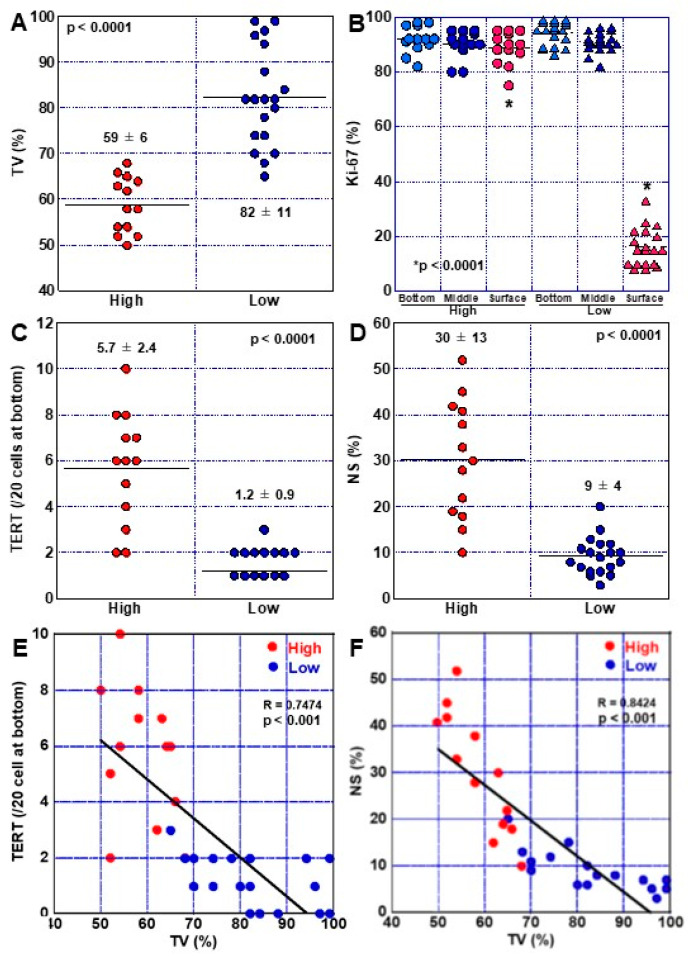
Comparison of high-grade and low-grade DM phenotypes. (**A**–**D**) TV (**A**), proliferation (**B**), TERT expression (**C**), and stemness (**D**) were compared between high-grade and low-grade DMs. (**E**) Relationship between TV and TERT expression. (**F**) Relationship between TV and stem cells. Data in panels, mean ± SD. DM, dysplastic metaplasia; High, high-grade DM; Low, low-grade DM; TV, telomere volume; TERT, telomerase reverse transcriptase; NS, nucleostemin; R, Pearson correlation coefficient.

High-grade and low-grade DMs were compared among the 32 DM cases (Figure 4). Our analysis showed that TV was significantly lower in high-grade DMs than in low-grade DMs (Figure 4A). We next compared the Ki67-positive ratio in the upper, middle, and lower regions of the gland duct. No significant difference was observed between the high-grade and low-grade cases in the lower and middle regions of the duct, but high-grade DMs showed a significantly higher Ki67-positive ratio in the upper portion of the duct than low-grade DMs (Figure 4B). The numbers of TERT-positive and NS-positive cells were also significantly increased in high-grade DMs compared with low-grade DMs (Figure 4C,D). p53 nuclear accumulation was found in two cases of high-grade DM, with less than 10% of the cells exhibiting low positive p53 expression (Table 3). We also observed an overall significant inverse correlation between TV and the proportion of TERT-positive cells in 32 cases of DM (Figure 4E), while TV and the NS-positive ratio also exhibited a significant inverse correlation (Figure 4F).

### 2.5. Progression of DM to Cancer

**Figure 5:**
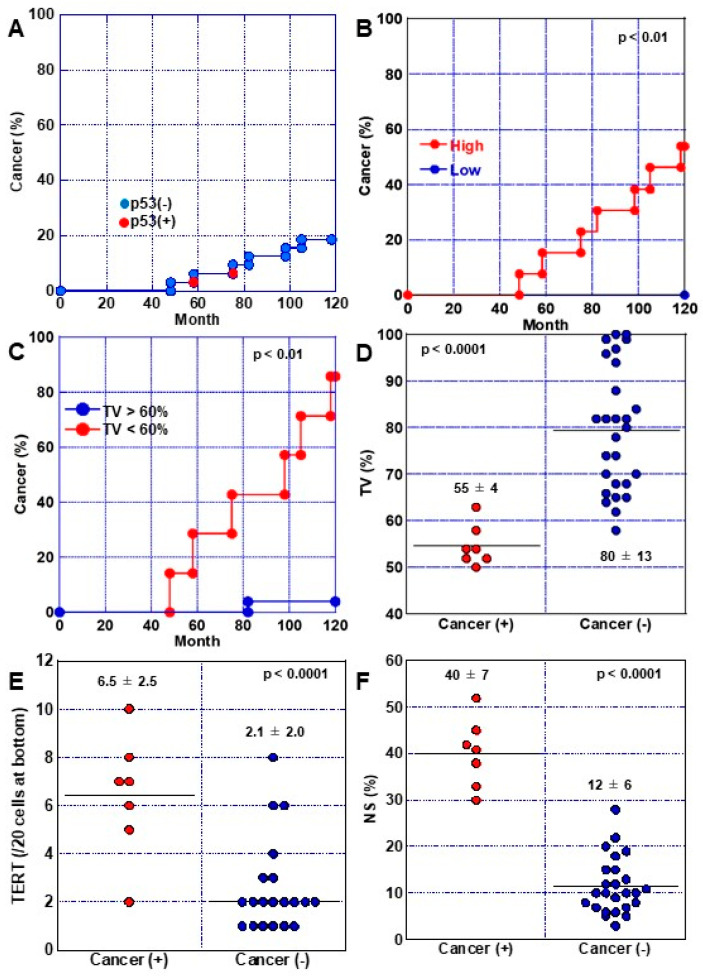
Comparison of DM phenotypes with/without cancer development. (**A**) Cancer development in 32 DMs. (**B**) Comparison of cancer development between high-grade and low-grade DMs. (**C**) Comparison of cancer development between lowTV (<60%) and high-TV (>60%) DMs. (**D**–**F**) TV (**D**), TERT expression (**E**) and stem cell number (**F**) were compared between DMs with/without cancer development. Data in panels, mean ± SD. DM, dysplastic metaplasia; High, high-grade DM; Low, low-grade DM; TV, telomere volume; TERT, telomerase reverse transcriptase; NS, nucleostemin.

To investigate the risk of developing gastric cancer in DM, the 32 DM cases were followed up for 10 years and non-invasive cancer developed in seven cases (22%) (Figure 5A). Cancers arising from all seven cases exhibited histology of well-differentiated tubular adenocarcinoma (tub1) localized in the mucosa, and all were positive for expression of p53 and caudal type homeobox transcription factor 2 (CDX2). Comparing cancer development by DM grade, 7 of the 13 high-grade DM cases developed cancer, which represents a carcinogenesis rate of 54% (Figure 5B). Examination of the relationship between TV and carcinogenesis revealed that TV was 60% or less in all but one case (Figure 5C). TV was significantly lower in cancer-developing cases, and the numbers of TERT-positive and NS-positive cells were significantly increased in these cases compared with non-cancer-developing cases (Figure 5D–F). In addition, both p53-positive cases progressed to cancer (Figure 5A).

### 2.6. Endoscopic Characteristics Associated with DM

**Figure 6:**
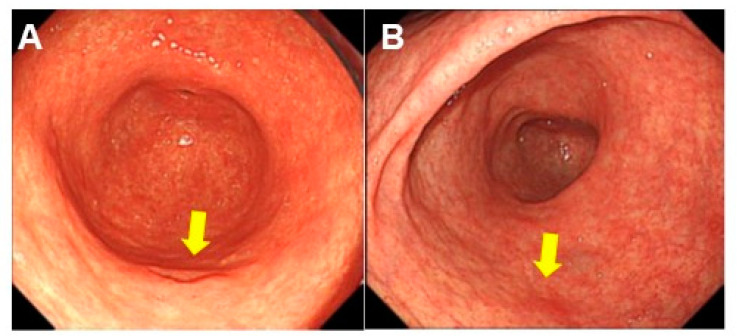
Endoscopic appearance of DM. (**A**) Case 9 in Table 3. High-grade DM. Male, 75 years old. Antrum, greater curvature. Lesion: 1.2 cm-sized depression with inflammation. (**B**) Case 3 in Table 3. Low-grade DM. Male, 72 years old. Lower corpus, greater curvature. Lesion: 0.8 cm-sized shallow depression with inflammation. Arrow, DM lesion. DM, dysplastic metaplasia.

As mentioned above, DM, especially high-grade DM, is associated with a high risk of carcinogenesis. Endoscopic detection of high-grade DM is significant in the prevention of gastric cancer. Therefore, we extracted and compared the endoscopic analyses of high-grade and low-grade DM (Figure 6 and Table 4).

DM sites were identified in almost the same number of locations in the antrum and the corpus of the stomach in both grades, and no significant difference was observed between the two. High-grade lesions were often larger than 1 cm across (mean 1.4 cm), whereas low-grade lesions were significantly smaller (mean 0.75 cm). Atrophic changes were common to both grades, but a significantly higher proportion of high-grade DMs showed severe atrophy corresponding to O-2 or O-3 of the Kimura-Takemoto classification [21]. The shape of the lesions showed depression accompanied by redness in both high-grade DM and low-grade DM, and there was no significant difference between the two. No polyp formation, erosion, or ulceration was observed in either grade. Based on these results, the endoscopic characteristics of high-grade DM are considered depressions with a diameter of more than 1 cm, located in the antrum or body of the stomach, against a background of highly atrophic changes.

## 3. Discussion

In this study, we identified focal mucosal lesions using telomere shortening as a marker and showed that they display histological features of DM. Furthermore, our analyses indicate that high-grade DM is associated with a high carcinogenic risk.

As DNA polymerase cannot completely copy the telomeric DNA at the ends of chromosomes, approximately 50 nucleotides are lost with each cell division, resulting in shortened telomeres [13]. Thus, telomere shortening occurs as a result of cell proliferation in normal aging and pathological states. In addition, telomere shortening ultimately brings cells to a growth limit threshold, causing them to undergo senescence [22]. In contrast, in tissue stem cells and cancer cells, telomere synthesis is retained by telomerase activity, resulting in suppression of telomere shortening and maintenance of telomere length [23]. This allows for unlimited proliferation in these cells. Telomerase activation is essential for cell immortalization and malignant transformation, as it stabilizes telomere length and thus bypasses the senescence barrier [14].

Previous studies on precancerous lesions have identified telomere shortening in high-risk mucosa of ulcerative colitis [24] and shown that telomere shortening and TERT expression in gastrointestinal metaplasia is associated with *H. pylori* infection [9]. Telomere shortening and telomerase activation have also been observed in precancerous lesions in leukemia [25], and telomere shortening is known to promote carcinogenesis in mouse cancer models [26]. These findings suggest that telomere shortening is an early event in carcinogenesis.

Our study showed a significant correlation between reduction of TV and increased TERT expression and stem cell numbers, suggesting that telomere shortening triggers the acquisition and increase of stemness. Furthermore, tissue stem cells already exhibit telomerase activity, and telomerase activation associated with carcinogenesis may indicate an increase in stem cells [27]. However, if cells with telomerase activity are derived from tissue stem cells, telomere length shortening should not be observed. Our data showing telomere shortening and telomerase activation suggest that telomere shortening may activate telomerase. Recently, TERT promoter mutations have been identified in more than 50 types of cancer, suggesting that this mutation may be the most common and earliest occurring genetic abnormality [28,29]. It is likely that telomere shortening and telomerase activation may occur as a result of mutations in the TERT promoter during the process of telomere shortening that accompanies the growth promotion associated with *H. pylori* infection. However, only 1% of gastric cancers reported to date have been shown to carry TERT promoter mutations [30]. Thus, a scenario in which tissue stem cells with shortened telomeres proliferate owing to excessive tissue regeneration associated with *H. pylori* infection cannot be denied. Further experimental studies are required to clarify the molecular mechanisms underpinning these cellular processes.

In our data, high-grade DM was associated with a high carcinogenic rate. High-grade DMs displayed an increase in TERT expression and stem cell marker-positive cells. TERT expression and telomerase activation are thought to promote stemness and epithelial–mesenchymal transition, as well as carcinogenesis [31]. Extremely shortened telomeres result in telomere fusion, causing chromosomal instability and carcinogenesis [32]. Carcinogenesis from telomere dysfunction requires other checkpoint defects as well, such as p53 mutations and DNA repair defects [33,34]. In our study, p53 accumulation was observed in all adenocarcinomas arising from high-grade DM, suggesting the presence of a genetic abnormality. Thus, the TERT expression and increased stem cell numbers seen in high-grade DM are likely to promote carcinogenesis. Furthermore, *H. pylori* infection is a pathological condition that affects a wide range of gastric mucosa, starting in the gastric antrum and spreading to the gastric corpus. However, it forms a localized cancer lesion. Our discovery of telomere-shortening lesions suggests that progressive rounds of proliferation and telomere shortening occur in critical regions of *H. pylori* infection, resulting in a focal lesion that develops into cancer. In our study, intestinal metaplasia with nuclear enlargement and decreased mucus production was observed against a background of atrophic mucosa, which we considered a histological feature of DM. In addition, we suggest that the degree of nuclear enlargement and occupation rate of glands with nuclear enlargement are valuable criteria for classifying high-grade and low-grade DM.

*H. pylori* infection is associated with multifocal atrophic gastritis, which frequently advances to intestinal metaplasia, occasionally to dysplasia, and rarely to carcinoma, and positions gastric dysplasia as a precancerous lesion [35]. In the 2019 edition of the WHO classification of tumors of the digestive system [36], gastric dysplasia is divided into low-grade and high-grade, and the histologic subtypes are divided into intestinal, foveolar, crypt, tubule neck, and serrated. In our histological analyses, our DM cases resembled intestinal dysplasia [37], but differed in the absence of nuclear elongation. In our review, adenomas and adenomatous ducts were not included among the DM cases. In addition, unlike high-grade dysplasia, no structural atypia was observed even in high-grade DM tissues. As gastric dysplasia also includes adenoma, the disease concept is unclear. Therefore, the Japanese Classification for Gastric cancer excludes the disease entity of gastric dysplasia [20].

High-grade gastric dysplasia is associated with a high risk of progression to cancer and cancer complications [38,39]. In our high-grade DM cases, 54% progressed to carcinogenesis within 10 years, which may indicate that these lesions represent a higher carcinogenic risk than gastric dysplasia. Although high-grade gastric dysplasia may be diagnosed as non-invasive cancer in Japan [40], in our study, three pathologists confirmed that our high-grade DMs could not be classified as cancer. Based on these findings, we hypothesize that the high-grade DM described in this study may be positioned as a novel entity of gastric dysplasia in the carcinogenic pathway outlined by Correa.

From a different point of view, intestinal metaplasia with basal gland atypia has been reported by Li et al. [41]. The prevalence of this phenotype is 2.8%, and 6.3% are p53 positive. In many post-eradication gastric cancers, intestinal metaplasia similar to atypical gastritis is observed around the cancer foci, with increased proliferative activity and an increase in the number of stem cell marker-positive cells [42]. This suggests that high-grade DM may display the morphological characteristics of high-risk mucosa after eradication, and further investigation is required.

Endoscopically, high-grade DM was recognized as lesions with a rough surface, normal-colored shallow depressions or low elevations less than 1 cm in diameter located in markedly atrophic mucosa. These findings are not highly specific and may require further diagnostic techniques for more accurate diagnosis. Magnifying endoscopy with narrow band imaging (ME-NBI) enables detailed observation of mucosal surface patterns and microvessel running patterns, and it is useful for the diagnosis of early gastric cancer [43]. We expect that the application of ME-NBI is likely to be useful in the diagnosis of high-grade DM in the future.

Currently, there are many entities regarding precancerous lesions of gastric cancer, and we discussed their relationship with DM. However, morphological or molecular pathological differences between the entities are not necessarily definitive at present. Regarding the DM, we were unable to sufficiently prove the relationship with other entities this time. It is necessary to conduct a more detailed examination of the differences with existing entities in the future. In this study, we examined only p53 as a genetic abnormality related to the progression of DM to cancer. Currently, gastric cancer is molecularly pathologically classified into four types [44]. In order to clarify the pathway of progression of DM to gastric cancer in more detail and to clarify the relationship with the four molecular types, comprehensive examination of genetic abnormalities is necessary in the future. This study will enable the extraction of new driver gene abnormalities in gastric cancer, and is expected to lead to new therapeutic targets.

## 4. Materials & Methods

### 4.1. Patients

We retrospectively analyzed a total of 587 patients with *H. pylori*-infected gastric mucosa who underwent endoscopic resection at the Miyoshi Central Hospital and were histopathologically diagnosed by the Department of Molecular Pathology, Nara Medical University, Japan, between 2005–2015. *H. pylori* infection was confirmed by either speculum, antigen, or breath testing of biopsy specimens. Cases in which *H. pylori* eradication was performed prior to testing were excluded. As written informed consent could not be obtained from the patients for their participation in the current study, all identifying information was removed from patient samples prior to analysis to ensure strict zprivacy protection (unlinked anonymization). All procedures were performed in accordance with the Ethical Guidelines for Human Genome/Gene Research enacted by the Japanese Government and with the approval of the Ethics Committee of Nara Medical University, Japan (approval number, 937, 10 January 2010).

### 4.2. Histological Analyses

Histological evaluation was performed using hematoxylin and eosin staining by three pathologists (RFT, HK, SK) based on the Japanese Classification for Gastric cancer [20].

### 4.3. Immunohistochemistry

Consecutive 4-μm thick sections were immunohistochemically stained using the immunoperoxidase technique described previously [45], with the primary antibodies listed in Table 5 and appropriate secondary antibodies (Medical and Biological Laboratories, Nagoya, Japan) (all 0.2 µg/mL). The tissue sections were then color-developed with diamine benzidine hydrochloride (Dako, Glostrup, Denmark) and counterstained with Meyer’s hematoxylin (Sigma-Aldrich Chemical Co., St. Louis, MO, USA). Staining indices were evaluated by examining 1000 epithelial cells, and the frequency of positive nuclear staining was determined. For p53 and NS, an index of more than 10% was considered to represent a “positive case”. TERT evaluation was performed by assessing 100 cells at the bottom of the glands, and a positive cell number per 20 cells was considered to represent TERT expression. 

### 4.4. Fluorescence In Situ Hybridization 

Telomere length was determined using FISH [9]. Sections (10 µm thick) of each specimen were mounted on ProbeOn slides (Fisher Scientific, Pittsburgh, PA) for ISH. The telomere repeat probe (TTAGGG)_4_ [46] was labeled with fluorescein isothiocyanate (FITC) on the 3’-end (Sigma, Ishikari, Japan). The probe was diluted to 20 µg/mL with probe hybridization solution (50% formamide [Sigma Chemical Co., St. Louis, MO, USA], 0.5 M NaCl, 5% polyethylene glycol 8000 [Sigma]). The specimens were dewaxed and dehydrated with xylene and 100% ethanol. Specimens hydrated in tris-buffered saline (Sigma) were digested with 0.2% pepsin-2 M HCl (Dako) for one hour at 37 °C and then subjected to RNase A (10 µg/µL, Takara Biomedicals, Tokyo, Japan) treatment at 37 °C for 10 min. The specimens were then heated with probe solution at 100 °C for 5 min, cooled to 4 °C for 15 min, and then maintained at 37 °C for 2 h. The specimens were rinsed five times with 1× standard sodium chloride/sodium citrate at 45 °C. 

### 4.5. Assessment of Telomere Volume 

Specimens hybridized with the (TTAGGG)_4_ probe were examined using a 520 nm wavelength for FITC fluorescence using a BZ-X710 All-in-One fluorescence microscope (KEYENCE, Osaka, Japan). Digital images were saved and processed to determine fluorescence intensity. Briefly, 100 nuclei were identified from the images of each specimen, and hybridization signals were scanned as inverted gray-scale images using NIH ImageJ software (version 1.52, National Institute for Health, Bethesda, WA, USA). The mean signal intensity and mean nuclear area of the pooled 500 nuclei were calculated. The mean intensity was divided by the mean nuclear area (µm^2^) to adjust for differences in the amounts of DNA of the identified nuclei. The resulting value was considered the representative telomere intensity of the tissue and was termed “telomere volume” to distinguish it from telomere length determined using Southern blot analysis. 

Telomere sequences in gastric cancer cells were similarly detected with the FITC-labeled microprobe, and TV was quantified via digital image analysis. 

The TV of biopsy specimens was then compared to that of the intramucosal lymphocytes (set to 100 and used the control), because the TVs in these cells were the largest and most constant of those obtained in specimens of normal tissue. 

### 4.6. Laser Microdissection

Laser microdissection was performed to identify and collect the nuclei of epithelial cells from those of non-epithelial cells such as lymphocytes, as described in our previous study [47]. Sections (10 µm thick) of each specimen were microdissected after methylene blue staining, and glands were examined via light microscopy. The target cells were captured using an Arcturus PixCell II-AS1 LASER microdissection system (Biomedical Equipment, Tokyo, Japan) according to the manufacturer’s instructions. Nuclei collected from 1000 cells were used for DNA extraction using TRI Reagent, according to the manufacturer’s instructions (Sigma).

### 4.7. Southern Blot Analysis

DNA (1 μg) was electrophoresed on a 0.8% agarose gel and transferred to nitrocellulose filters using Southern blotting [48,49]. Filters were fixed for 2 h at 80 °C under vacuum and hybridized with FITC-labeled (TTAGGG)_4_ probes. After hybridization, the filters were washed under stringent conditions, and signal intensity was quantified by digital image analysis using ImageJ.

### 4.8. Slot Blot Analysis

DNA (0.2 μg) in 5 μL of TBS was slot blotted on to nitrocellulose filters using BioDotSF (BioRad, Hercules, CA, USA) [50]. The membrane was dried and fixed by UV crosslinking (BLX-Multichannel Bio-Link crosslinker, VILBER LOURMAT, Marne-la-Vallée, France). Filters were then hybridized with FITC-labeled (TTAGGG)_4_ probes. After hybridization, the filters were washed under stringent conditions, and signal intensity was quantified by digital image analysis using ImageJ.

### 4.9. Statistical Analysis

Statistical significance was calculated using a two-tailed Fisher’s exact test and ordinary ANOVA by using InStat software (version 3.0, GraphPad, Los Angeles, CA, USA). Regression analysis was performed using a Pearson’s regression test. Statistical significance was set at a two-sided *p* value < 0.05.

## 5. Conclusions

**Figure 7:**
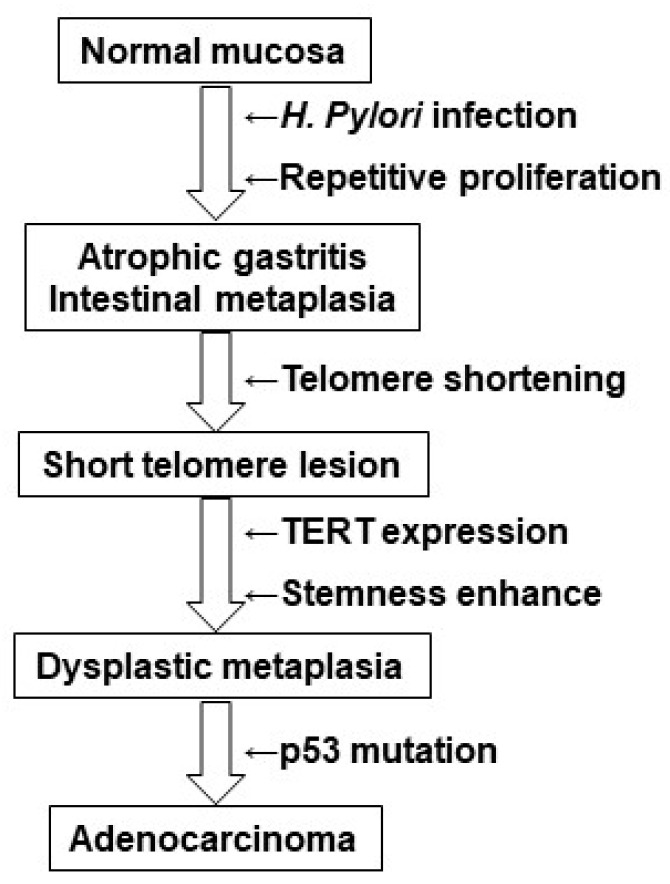
DM in *H. pylori*-associated gastric carcinogenesis pathway. The position of DM in the *H. pylori*-associated gastric carcinogenesis pathway is illustrated. See Conclusion section for explanation. DM, dysplastic metaplasia.

Our present study suggests an *H. pylori*-associated carcinogenic pathway as shown in Figure 7. Repetitive tissue destruction and regenerative proliferation in response to *H. pylori* infection results in atrophic gastritis/intestinal metaplasia. Acceleration of this regenerative proliferation process results in shortening of telomeres and formation of short telomere lesions. This triggers TERT expression and stem cell proliferation (or enhanced stemness), resulting in dysplastic metaplasia. This process may include the emergence of TERT gene promoter mutations. In addition, the progression from low-grade to high-grade DM may also involve gene mutations, in addition to differences in the degree of TERT expression and stemness. Finally, the addition of a p53 gene mutation may trigger the progression of DM to adenocarcinoma. Whether gene mutations other than p53 are involved in this stage is a question for future investigation.

We identified DM via our search for focal lesions using reduced TV as a marker in patients infected with *H. pylori*. High-grade DM is associated with a high carcinogenic rate and may represent a highly specific precancerous lesion in *H. pylori*-associated carcinogenesis. Identifying high-risk groups from *H. pylori*-infected gastric mucosa would have a significant impact on the prevention of gastric cancer, and a large-scale prospective study will be necessary to explore the value of this approach.

## Figures and Tables

**Table 1:** Short telomere lesion in five cases of gastric cancer.

Case	Cancer		Short Telomere Lesion			
	Histology ^(1)^	TV (%)	Histology	Grade	TV (%) ^(2)^	TERT
1	tub2, pT1 (lesion 1, area I)	43	IM (around cancer)	Low	65	5
	tub2 + por2, pT3 (lesion 2, area IV)	32	IM (around cancer)	High	55	10
			IM (area II)	High	58	8
			IM (area III)	Low	66	3
2	tub1, pT1	45	IM (around cancer)	High	58	8
			IM	Low	75	2
3	tub1, pT1 (area I)	47	IM (area II)	Low	72	1
4	tub1, pT1	51	IM (around cancer)	Low	75	3
			IM	Low	72	2
5	tub2, pT1	42	IM (around cancer)	Low	68	2
			IM	High	61	6

^(1)^ Histology and invasive depth were classified in according to Japanese Classification of Gastric Cancer [20]. Tub1, well-differentiated tubular adenocarcinoma; tub2, moderately differentiated tubular adenocarcinoma; por2, poorly differentiated adenocarcinoma, non-solid type; pT1, Tumor confined to the mucosa or submucosa; pT3, tumor invades the subserosa (SS). ^(2)^ TV is noted as a relative value when the TV value of intramucosal infiltrating lymphocytes is 100%. TV, telomere volume; TERT, telomerase reverse transcriptase; IM, intestinal metaplasia.

**Table 2:** Histological diagnostic criteria of high-grade and low-grade DMs.

Histological Feature	Low-Grade DM	High-Grade DM
Nuclear enlargement	Less than twice the size of lymphocyte nucleus	More than twice the size of lymphocyte nucleus
Area of nuclear enlargement	Less than 1/3 of gland base	More than 1/3 of gland base
Structural atypia	None	None
Mucus production	Reduced: mild to moderate	Reduced: moderate to marked
Goblet cell	Reduced: mild to moderate	Reduced: moderate to marked

* CDX2, caudal type homeobox transcription factor 2; NS, nucleostemin. ^†^ Dako, Santa-Clara, CA, USA; Santa Cruz Biotechnology, Santa-Cruz, CA, USA; Abcam, Cambridge, UK.

**Table 3:** Thirty two cases of DM out of 587 *H. pylori*-positive gastric mucosa cases.

Case	Grade	TV (%)	Ki-67	(%) *		TERT ^†^	NS	p53	Months to Cancer
			Lower	Middle	Upper		(%)	(%)	diagnosis
1	Low	88	89	90	24	0	8	0	-
2	Low	99	92	90	15	0	5	0	-
3	Low	82	95	96	20	1	6	0	-
4	High	62	91	92	88	3	15	0	-
5	Low	74	96	95	25	2	12	0	-
6	Low	68	88	90	33	2	13	0	-
7	High	66	97	95	82	4	18	0	-
8	High	68	82	80	83	2	10	0	-
9	Low	70	99	95	22	1	11	0	-
10	High	52	98	92	95	5	45	5	58
11	High	65	92	90	88	6	22	0	-
12	High	50	85	80	75	8	41	8	75
13	Low	96	86	88	10	1	5	0	-
14	Low	94	98	92	8	2	7	0	-
15	Low	97	95	96	15	0	3	0	-
16	Low	99	99	94	10	2	7	0	-
17	High	64	98	95	90	6	19	0	-
18	High	54	90	88	92	6	52	0	98
19	Low	82	95	92	16	0	10	0	-
20	High	58	89	90	87	7	38	0	105
21	High	58	97	94	90	8	28	0	-
22	High	63	92	95	94	7	30	0	82
23	Low	82	98	92	9	2	10	0	-
24	High	52	92	90	95	2	42	0	48
25	Low	78	88	82	18	2	15	0	-
26	Low	82	98	90	15	1	10	0	-
27	Low	84	99	91	10	0	8	0	-
28	Low	80	93	88	8	1	6	0	-
29	High	54	92	90	95	10	33	0	118
30	Low	65	95	91	22	3	20	0	-
31	Low	70	97	89	15	2	9	0	-
32	Low	74	89	85	10	1	12	0	-

DM, dysplastic metaplasia.

**Table 4:** Endoscopic analyses of DM.

Endoscopical Finding	Incidence	(%)	*p* Value
Grade	High grade	Low grade	
Age (yo)	76 ± 12	70 ± 13	NS
Sex (male:female)	9:4	12:7	NS
Location			NS
Antrum	7	9	
Corpus	6	10	
Cardia	0	0	
Size			<0.0001
<1cm	1	15	
>1cm	12	4	
Atrophy *			0.0007
C-3 or O-1	0	11	
O-2 or O-3	13	8	
Shape			NS
Depression	10	14	
Elevation	3	3	
Flat	0	2	
Polyp	0	0	
Erosion	0	0	
Ulcer	0	0	
Color			NS
Normal	3	10	
Redness	10	9	

* Endoscopic classification of atrophic gastritis followed the Kimura-Takemoto classification [21]. C-3, border of atrophy lying on the lesser curvature in the middle third of the gastric corpus; O-1, border of atrophy involving the boundary lying between the lesser curvature and the anterior wall of the gastric corpus; O-2, border of atrophy lying within the anterior wall of the gastric corpus; and O-3, border of atrophy involving the boundary between the posterior wall of the gastric corpus and large curvature. NS, nucleostemin.

**Table 5:** Antibodies.

Target *	Manufacturer ^†^	Clone	Code	Working Concentration (μg/mL)
Ki-67	Dako	MIB-1	M7240	0.5
TERT	Santa-Cruz	A-6	sc-393013	1
CDX2	Abcam	CDX2-88	ab157524	0.2
p53	Abcam	PAb240	ab26	1
NS	Abcam	-	ab70346	0.5

* CDX2, caudal type homeobox transcription factor 2; NS, nucleostemin. ^†^ Dako, Santa-Clara, CA, USA; Santa Cruz Biotechnology, Santa-Cruz, CA, USA; Abcam, Cambridge, UK.

## Data Availability

Not applicable.

## Abbreviations

STL: short telomere lesions; DM, dysplastic metaplasia; TERT, telomerase reverse transcriptase; FISH, fluorescence in situ hybridization; TV, telomere volume; NS, nucleostemin; CDX2, caudal type homeobox transcription factor 2.
